# Supplementary material for: Context congruence: How associative learning modulates cultural evolution
Source: PLoS One. 2023 Apr 4;18(4):e0282776. doi: 10.1371/journal.pone.0282776 (PMC10072484; doi:10.1371/journal.pone.0282776)
Supplement: S1 File — (DOCX) [file pone.0282776.s001.docx]

Supplementary Materials: Context congruence: How associative learning modulates cultural evolution

# Experiment design

In order to control our confounding variables, we counterbalance the strategy variant – Parity (strategy A) and Skipping (strategy B) – and the order of the presentation of the two strategies to the participants (expert’s first and peer’s first). Participants were allocated to one of two subgroups, so that each subgroup had sixteen participants (Fig. SM_1):

*
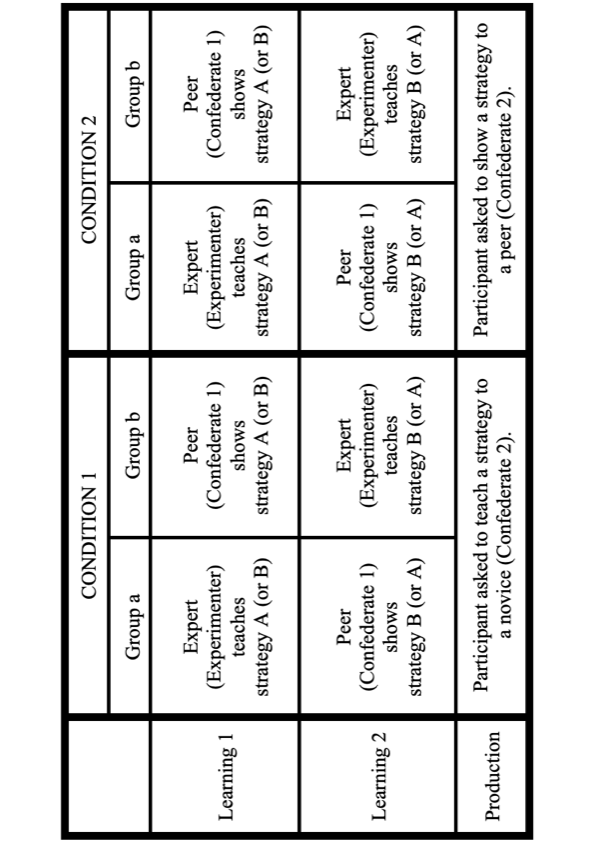
*

Figure SM_1. The two conditions and their subgroups. In the production phase, in Condition 1, the participant assumes the role of an expert, and is asked to teach a strategy to a novice; in condition 2, the participant assumes the role of a peer, and is asked to show a strategy to a peer. In each group, the identity and order of strategy variants are fully counterbalanced.

- Expert-Peer-Novice: All sixteen participants in this group were asked to teach a novice (a confederate) how to solve the problem. Eight of the participants in this group were first taught by the expert strategy A, and then they were shown by a peer strategy B. The remaining eight participants were first taught by the expert strategy B, and then they were shown by a peer strategy A.
- Peer-Expert-Novice: All sixteen participants in this group were asked to teach a novice (a confederate) how to solve the problem. Eight of the participants in this group were first shown by a peer strategy A, and then they were taught by the expert strategy B. The remaining eight participants were first shown by a peer strategy B, and then they were taught by the expert strategy A.
- Expert-Peer-Peer: All sixteen participants in this group were asked to show a peer (a confederate) how to solve the problem. Eight of the participants in this group were first taught by the expert strategy A, and then they were shown by a peer strategy B. The remaining eight participants were first taught by the expert strategy B, and then they were shown by a peer strategy A.
- Peer-Expert-Peer: All sixteen participants in this group were asked to show a peer (a confederate) how to solve the problem. Eight of the participants in this group were first shown by a peer strategy A, and then they were taught by the expert strategy B. The remaining eight participants were first shown by a peer strategy B, and then they were taught by the expert strategy A.

# Experiment procedure by condition (Dialogues)

## Expert’s strategy first, participant teaches novice:

### Phase 1: Learning

- Experimenter – I will teach you the solution I’ve taught many people before as part of my experiment*. [Teaches strategy].*
- C1 (Peer) – I’ve played this before, I know another solution. *[Shows alternative strategy].*
- Experimenter – *[Initiates Phase 2]:* Oh okay, that’s interesting! So now you both know two different solutions to this problem. Okay, for the next part I need you both to teach your solution to two other participants who don’t know how to solve the problem, yet. So, I’ll need one of you in this lab and the other one to the other lab. Who would like to come with me to the other lab?
- C1 (Peer) – I’ll come.
- The experimenter leads C1 out and, after approximately ten seconds, brings in a different confederate (C2): the novice.

### Phase 2: Onward Transmission

- Experimenter – *[Looks at C2]* Okay so, the problem is to figure out which way the last gear turns… [Participant] is now an expert at this, and he/she will now teach you his/her solution to the problem.

## Peer’s strategy first, participant teaches novice:

### Phase 1: Learning

- C1 (Peer) – I’ve played this before, I know a solution. *[Shows strategy].*
- Experimenter – Oh okay, that’s interesting! I will also teach you the solution I’ve taught many people before as part of my experiment. *[Teaches alternative strategy, then initiates Phase 2].*
- The experimenter leads C1 out and, after approximately ten seconds, brings in a different confederate (C2): the novice.

### Phase 2: Onward Transmission

- Experimenter – *[Looks at C2]* Okay so, the problem is to figure out which way the last gear turns… [Participant] is now an expert at this, and he/she will now teach you his/her solution to the problem.

## Expert’s strategy first, participant shows to peer:}

### Phase 1: Learning

- Experimenter – I will teach you the solution I’ve taught many people before as part of my experiment. *[Teaches strategy].*
- C1 (Peer) – I’ve played this before, I know another solution. *[Shows alternative strategy. Experimenter initiates Phase 2; experimenter leads C1 out and, after approximately ten seconds, brings in a different confederate (C2): the peer]*

### Phase 2: Onward Transmission

- Experimenter – *[Looks at C2]* Okay so, the problem is to figure out which way the last gear turns… [Participant] is a participant just like you, and he/she will show you his/her solution to the problem.

## Peer’s strategy first, participant shows to peer:

### Phase 1: Learning

- C1 (Peer) – I’ve played this before, I know a solution. *[Shows strategy].*
- Experimenter – Oh okay, that’s interesting! I will also teach you the solution I’ve taught many people before as part of my experiment. *[Experiment teaches alternative strategy, then initiates Phase 2. The experimenter leads C1 out and, after approximately ten seconds, brings in a different confederate (C2): the peer].*

### Phase 2: Onward Transmission

- Experimenter – *[Looks at C2]* Okay so, the problem is to figure out which way the last gear turns… [Participant] is a participant just like you, and he/she will show you his/her solution to the problem.

# How *ExpertBias*, *CongruenctBias* and *PrimacyBias* influence the probabilities that the different variants are selected in the simulations

Table SM_1. Calculation of the probabilities that the congruent and incongruent variants are produced in each experimental condition, as a function of Expert, Congruent and Primacy biases. In Italics, number of participants in each condition, and count of variants they produced.

|  | Condition: Production context | | | |
| --- | --- | --- | --- | --- |
|  | Variant produced was learned First in the context below  *Experiment, 30 participants* | | Variant produced was learned Second in the context below  *Experiment, 32 participants* | |
|  | Expert-to-novice  *Experim = 18 partic.* | Peer-to-peer  *Experim = 12 partic.* | Expert-to-novice  *Experim = 23 partic.* | Peer-to-peer  *Experim = 9 partic.* |
| Probability to produce Congruent variant | ExpertBias x CongruentBias x PrimacyBias  *Experim. count = 12* | (1 - ExpertBias) x CongruentBias x PrimacyBias  *Experim. count = 2* | ExpertBias x CongruentBias x (1 - PrimacyBias)  *Experim. count = 15* | (1 - ExpertBias) x CongruentBias x (1 - PrimacyBias)  *Experim. count = 1* |
| Probability to produce Incongruent variant | ExpertBias x (1 - CongruentBias) x PrimacyBias  *Experim. count = 6* | (1 - ExpertBias) x (1 - CongruentBias) x PrimacyBias  *Experim. count = 10* | ExpertBias x (1 - CongruentBias) x (1 - PrimacyBias)  *Experim. count = 8* | (1 - ExpertBias) x (1 - CongruentBias) x (1 - PrimacyBias)  *Experim. count = 8* |

Figure SM_2. Average number of simulation runs (out of *S* = 1000), collapsing all values of Expert Bias and Congruence Bias, that matched the experimental data with different values of Primacy Bias.
